# AI-Driven Comprehensive SERS-LFIA System: Improving Virus Automated Diagnostics Through SERS Image Recognition and Deep Learning

**DOI:** 10.3390/bios15070458

**Published:** 2025-07-16

**Authors:** Shuai Zhao, Meimei Xu, Chenglong Lin, Weida Zhang, Dan Li, Yusi Peng, Masaki Tanemura, Yong Yang

**Affiliations:** 1 State Key Laboratory of High Performance Ceramics, Shanghai Institute of Ceramics, Chinese Academy of Sciences, 1295 Dingxi Road, Shanghai 200050, China; 2 Center of Materials Science and Optoelectronics Engineering, University of Chinese Academy of Sciences, Beijing 100049, China; 3 Graduate School of the Chinese Academy of Sciences, No.19 (A) Yuquan Road, Beijing 100049, China; 4 Department of Frontier Materials, Nagoya Institute of Technology, Nagoya 466 8555, Japan; tanemura.masaki@nitech.ac.jp

**Keywords:** automated detection system, SERS-LFIA, machine learning, deep learning, SARS-CoV-2

## Abstract

Highly infectious and pathogenic viruses seriously threaten global public health, underscoring the need for rapid and accurate diagnostic methods to effectively manage and control outbreaks. In this study, we developed a comprehensive Surface-Enhanced Raman Scattering–Lateral Flow Immunoassay (SERS-LFIA) detection system that integrates SERS scanning imaging with artificial intelligence (AI)-based result discrimination. This system was based on an ultra-sensitive SERS-LFIA strip with SiO_2_-Au NSs as the immunoprobe (with a theoretical limit of detection (LOD) of 1.8 pg/mL). On this basis, a negative–positive discrimination method combining SERS scanning imaging with a deep learning model (ResNet-18) was developed to analyze probe distribution patterns near the T line. The proposed machine learning method significantly reduced the interference of abnormal signals and achieved reliable detection at concentrations as low as 2.5 pg/mL, which was close to the theoretical Raman LOD. The accuracy of the proposed ResNet-18 image recognition model was 100% for the training set and 94.52% for the testing set, respectively. In summary, the proposed SERS-LFIA detection system that integrates detection, scanning, imaging, and AI automated result determination can achieve the simplification of detection process, elimination of the need for specialized personnel, reduction in test time, and improvement of diagnostic reliability, which exhibits great clinical potential and offers a robust technical foundation for detecting other highly pathogenic viruses, providing a versatile and highly sensitive detection method adaptable for future pandemic prevention.

## 1. Introduction

Highly pathogenic and contagious diseases, including severe acute respiratory syndromes (SARS), Middle East respiratory syndrome (MERS), and the recent global pandemic of coronavirus disease 2019 (COVID-19), continue to challenge global public health infrastructure. The causative agent of COVID-19, severe acute respiratory syndrome coronavirus-2 (SARS-CoV-2), has demonstrated exceptional transmissibility and pathogenicity, leading to unprecedented morbidity, mortality, and socioeconomic disruption worldwide [1]. While the immediate threat of SARS-CoV-2 has reduced, the persistent emergence and re-emergence of infectious viruses underscore the critical need for robust and rapid diagnostic tools. Timely and accurate viral detection is paramount for effective clinical management, outbreak containment, and minimizing societal burden. Furthermore, advancing such technologies is essential for bolstering preparedness against future epidemic threats.

In response to the diagnostic demands highlighted by the COVID-19 pandemic, diverse methodologies have been deployed for viral detection [2,3]. Reverse transcription polymerase chain reaction (RT-PCR) remains the benchmark for sensitivity, capable of identifying minute quantities of viral RNA. However, the practical utility of RT-PCR is constrained by its reliance on specialized laboratory infrastructure, skilled personnel, and lengthy processing times (often exceeding several hours), rendering it less ideal for rapid, point-of-care testing (POCT) or mass testing scenarios. Conversely, lateral flow immunoassays (LFIAs) targeting viral antigens offer a significantly faster and more accessible alternative. LFIA devices can deliver results typically within 15–30 min and are operable outside conventional laboratory settings, making them suitable for widespread deployments. Tong et al. [4] developed an AI-assisted colorimetric LFIA platform, utilizing polydopamine nanoparticles for neutralizing antibody detection (LOD 160 ng/mL), demonstrating the potential for enhanced LFIA performance. However, the comparatively lower sensitivity has always been a crucial factor restricting the further development of conventional LFIA technology, particularly at low viral loads, increasing the risk of false negative outcomes.

To bridge the sensitivity gap inherent in traditional LFIA, various advanced signal transduction strategies have been integrated, including electrochemical [5], fluorescence [6], photothermal [7], and surface-enhanced Raman scattering (SERS) [8]. SERS stands out for its exceptional molecular fingerprinting capability and potential for single-molecule detection [9]. Recent progress in SERS-LFIA for SARS-CoV-2 detection has largely focused on optimizing nanoprobe to achieve lower LODs. Generally, nanoprobes with stronger SERS signal amplification ability produced higher assay sensitivity [10]. Examples include Liu et al.’s magnetic SERS tags achieving an impressive theoretical LOD of 8 pg/mL [11] and our previous work utilizing Ag/black phosphorus (Ag/BP) nanocomposites for rapid and sensitive variant detection with a portable Raman system [12]. Despite the fact that research on the sensitivity of the SERS-LFIA system mentioned above has been improved, practical implementation faces significant hurdles: (1) Complex nanoprobe synthesis and stability issues that affect the long-term usability and practicality; and (2) Signal heterogeneity arising from inherent variations in nitrocellulose (NC) membrane composition and the stochastic nature of point-based SERS sampling. This heterogeneity often leads to localized intensity anomalies, especially near the detection limit, adversely impacting result accuracy and reliability.

**Figure 1:**
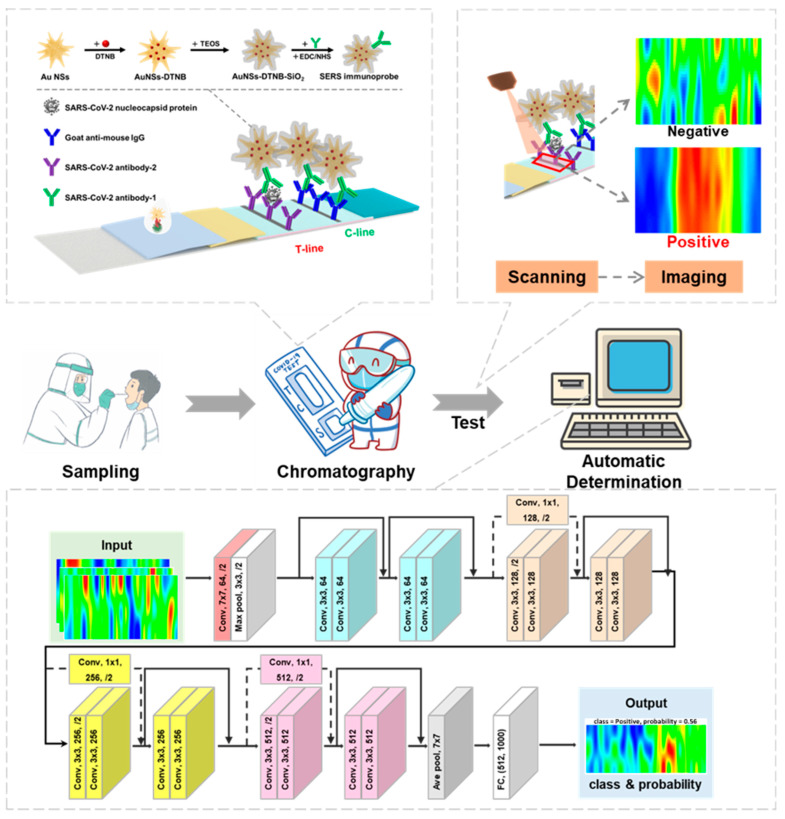
Schematic of the SARS-CoV-2 detection and classification process including working principles of the Au NSs-DTNB-SiO_2_ immunoprobe-based SERS-LFIA strip; the principle of SERS scanning imaging and the architecture of the ResNet-18 image recognition model.

Addressing these critical challenges, a novel and ultra-sensitive SERS-LFIA system specifically engineered for SARS-CoV-2 detection that integrates large-area SERS imaging with deep learning-based analysis was presented. The developed SERS-LFIA system distinguishes itself through a synergistic combination of high sensitivity, specificity, and automation, positioning it as an ideal POCT protocol. As depicted in Figure 1, the core innovation of this work lies in the use of a deep residual neural network (ResNet-18) model to analyze the spatial distribution patterns of nanoprobes across the T line captured via SERS scanning imaging, which is different from the conventional SERS-LFIA methods that rely solely on Raman intensity measurements at random points on the T line. This method effectively reduces the interference of abnormal signals, significantly improves the classification accuracy, and can achieve fast, independent, and automatic detection, which is suitable for POCT environment. Collectively, these advancements establish our integrated AI-driven SERS-LFIA platform as a transformative and broadly applicable tool for the rapid, reliable detection of SARS-CoV-2 and other highly pathogenic pathogens.

## 2. Materials and Methods

### 2.1. Materials

Gold chloride trihydrate (HAuCl_4_·3H_2_O, ≥99.9%), Sodium citrate dihydrate (99.0%), Silver nitrate (AgNO_3_, 99.8%), Hydrochloric acid (HCl, 37.0%), Ascorbic acid (AA, 99.0%), 5,5′-Dithio bis-(2-nitrobenzoic acid) (DTNB, 98.0%), Sodium hydroxide (NaOH, ≥98.0%), (3-Mercaptopropyl) trimethoxy silane (MPTS, 95.0%), Tetraethyl orthosilicate (TEOS, 99.99%), (3-Aminopropyl) triethoxysilane (APTES, 99.99%), Tris-(hydroxymethyl)-aminomethane (Tris, 99.9%), Triton™ X-100 (Triton), Polyvinylpyrrolidone (PVP, k30), Ethylenediaminetetraacetic acid (EDTA, 98.0%), TWEEN-20, and Casein were purchased from Shanghai Aladdin Biochemical Technology Co., Ltd. (Shanghai, China). 4-Morpholineethanesulfonic acid (MES), N-(3-Dimethylaminopropyl)-N′-ethyl carbodiimide hydrochloride (EDC), N-Hydroxy succinimide (NHS), Phosphate buffered saline (PBS) and bovine serum albumin (BSA, 96.0%) were sourced from Sigma-Aldrich (St. Louis, MO, USA). Nitrocellulose (NC) membrane (CN140) was purchased from Sartorius (Göttingen, Germany). Fetal Bovine Serum (FBS) and Dulbecco’s Modified Eagle Medium (DMEM) was obtained from Gibco (Grand Island, New York, USA). Other components of chromatography strips, including the sample pad, the absorbent pad, and polyvinyl chloride (PVC) plate, were purchased from Shanghai Jieyi Biotechnology Co., Ltd. (Shanghai, China). Block agent (HIER-R-001), Goat anti-mouse IgG, SARS-CoV-2 antibody (COVID-19-PS-MAb1), SARS-CoV-2 antibody (COVID-19-PS-MAb2), H1N1 antigen, Influenza A antigen (FluA), Influenza B antigen (FluB), and human respiratory syncytial virus (HRSV) were produced from Fapon Biotechnology Co., Ltd. (Dongguan, Guangdong, China). SARS-CoV-2 nucleocapsid protein (NP) was purchased from Beijing Sino Biological, Inc. (Beijing, China). All chemicals were used without further purification.

### 2.2. Synthesis of Au NSs and Au NSs-DTNB-SiO_2_ Immunoprobe

**Synthesis of Au NSs:** Au NSs were prepared with reference to our previous work [13] with some adjustments. Initially, 100 mL of 1 mM HAuCl_4_ solution was heated to boiling in an oil bath. Once boiling, 4 mL of 1 wt% sodium citrate solution was added, and the solution was kept boiling for an additional 15 min under continuous mechanical stirring. After cooling to room temperature, Au seeds were obtained. For the subsequent growth process, 100 μL of 1 M HCl and 1 mL of the prepared Au seeds were added to 100 mL solution of 0.25 mM HAuCl_4_. The mixture was stirred continuously at room temperature, followed by the addition of 600 μL of 1 mM AgNO_3_ solution and 500 μL of 100 mM AA solution. The reaction was stirred for 30 min, resulting in the formation of Au NSs.

**Preparation of Au NSs-DTNB-SiO_2_:** DTNB is a widely used Raman reporter. Its peak shape is simple and it contains -SH, which can combine with gold nanomaterials to form stable Au-SH bonds. A total of 1 mL of DTNB solution (10^−4^ M) and 50 μL of MPTS (2.5 × 10^−5^ M) were simultaneously added to the 100 mL Au NSs solution. The mixture was sonicated for 15 min to ensure dispersion and then stirred for an additional 30 min. The pH of the colloidal Au NSs solution was adjusted to about 9.5 using NaOH. To coat the Au NSs with a silica layer, 40 μL of TEOS (20 wt%) was added to the solution every 30 min with continuous stirring. This process was repeated three times, and the mixture was stirred for an additional 24 h to ensure the formation of the silica shell. The resulting Au NSs-DTNB-SiO_2_ composite was washed with deionized (DI) water and ethanol before being dispersed in 40 mL of ethanol for future use.

**Synthesis of Au NSs-DTNB-SiO_2_ immunoprobes:** The Au NSs-DTNB-SiO_2_ solution was transferred to a three-necked flask and heated to 60 °C with constant stirring. Then, 1 mL of APTES was added to the solution, and the reaction proceeded for 12 h, resulting in amino-functionalized Au NSs-DTNB-SiO_2_ (Au NSs-DTNB-SiO_2_-NH_2_).

To collect the desired product, 3 mL of the Au NSs-DTNB-SiO_2_-NH_2_ solution was centrifuged at 9567 g, and the lower layer was isolated. This material was washed with DI water and subsequently dispersed in 500 μL of MES buffer solution (0.01 M). The conjugation of the SARS-CoV-2 antibody (COVID-19-PS-MAb1) to the Au NSs-DTNB-SiO_2_-NH_2_ was achieved through EDC/NHS activation. Specifically, 15 μg of the antibody, 5 μL of EDC (1 mM), and 10 μL of NHS (2 mM) were added to the solution and shaken for 1.5 h at room temperature to form stable amide bonds between the antibody’s carboxyl groups and the amino groups on the functionalized Au NSs-DTNB-SiO_2_-NH_2_.

To minimize nonspecific binding during subsequent experiments, 100 μL of the blocking solution containing 5% BSA and 5% casein was added to the immunoprobe solution. This mixture was shaken at 25 °C for 1 h. After the blocking step, the immunoprobes were centrifuged at 9567 g to collect the precipitate, which was washed with 500 μL of 10 mM PBST (pH 7.4, 0.05% Tween-20). Finally, the immunoprobes were resuspended in 200 μL of storage solution (10 mM PBS, 0.05% Tween-20, 0.5% BSA, and 0.02% NaN_3_) and stored at 4 °C for future use.

### 2.3. Fabrication of LFIA Strip

**Preparation of the C and T Lines:** For the LFIA strip fabrication, goat anti-mouse IgG (1 mg/mL) and SARS-CoV-2 antibody (COVID-19-PS-MAb2) (1.2 mg/mL) were used for the C line and T line, respectively. These antibodies were dispensed onto the NC membrane using a Biodot XYZ3050 3D plotter, with a dispensing speed of 0.1 μL/mm. Following deposition, the membrane strips were dried in an incubator at 37 °C overnight to ensure proper antibody immobilization.

**Blocking the sample pad:** The glass fiber sample pad was treated with a blocking agent (HIER-R-001) to reduce nonspecific binding during the assay. The blocking solution was prepared by diluting HIER-R-001 with Tween-20 to a final concentration of 0.4 mg/mL. This solution was sprayed onto the sample pad using the same plotter. The treated sample pad was dried overnight at 37 °C in an incubator to ensure thorough absorption and uniform distribution of the blocking reagent.

**Deposition of Immunoprobes on the Conjugate Pad:** The prepared Au NSs-DTNB-SiO_2_ immunoprobes were then applied to the conjugate pad using the same equipment at a dispensing speed of 1.5 μL/mm. After deposition, the conjugate pads were dried in an incubator at 37 °C overnight to stabilize the immunoprobes on the pad surface.

**Assembly of the LFIA Strip:** Once the individual components were prepared, the LFIA strip was assembled by sequentially affixing the sample pad, NC membrane (with C and T lines), and a conjugate pad onto a PVC backing board. This assembly was carefully aligned to ensure proper flow of the sample through the strip during testing. The fully assembled strips were then cut into 3 mm wide sections and stored under controlled conditions for future use.

### 2.4. Detection of SARS-CoV-2 NP

To assess the sensitivity of the SERS-LFIA system based on Au NSs-DTNB-SiO_2_ immunoprobes, varying concentrations of SARS-CoV-2 NP were tested. The NP solution was diluted using a lysis buffer composed of 0.1 M Tris (pH 8.2), 2% Triton, 1% PVP, and 0.01 M EDTA, with final concentrations ranging from 10,000 pg/mL to 0.1 pg/mL. For each test, 100 μL of the diluted SARS-CoV-2 NP solution was applied directly to the sample pad of the LFIA strip. The immune reaction was allowed to proceed for 12 min, after which the strip was removed and dried for 5 min. Following this, SERS spectra or SERS imaging was performed using a portable Raman spectrometer from Oceanhood (RMS-2000, Excitation wavelength: 785 ± 0.5 nm, spectral resolution: ~8 cm^−1^@25 μm, laser power: 0–500 mW). For Raman spectral analysis, the laser power was set to 100 mW with an integration time of 2 s. During SERS mapping, a rectangular scanning area of 1.8 mm × 0.5 mm cantered on the T line was selected, and the signal was analyzed based on the peak area in the range of 1300–1350 cm^−1^. The laser power was reduced to 50 mW with an integration time of 0.1 s, and the laser was set to ‘always on’ mode to ensure consistent illumination throughout the mapping process.

### 2.5. Signal Recognition Based on SERS Imaging

After the completion of chromatography and subsequent drying of the LFIA strip, Raman signals are detected along and around the T line using a portable Raman spectrometer. The imaging process involves integrating the peak area of the strongest Raman characteristic signal, typically within the range of 1300–1350 cm^−1^, corresponding to the probe molecule. By analyzing the resulting image and assessing variations in the Raman signal intensity, the presence of viral antigens at the T line can be determined. The specific procedure is outlined below.

#### 2.5.1. Imaging Area Specifications

The imaging area is defined as a rectangular region with dimensions 1 mm ≤ X ≤ 3 mm and 0.2 mm ≤ Y ≤ 3 mm, centered geometrically on the T line. In this setup, the X-direction corresponds to the direction of liquid flow, while the Y-direction aligns with the strip label direction. The preferred dimensions for this work are X = 1.8 mm and Y = 0.5 mm.

#### 2.5.2. Scanning Step Size Control

During the scanning process, we control the scanning step size in the X direction, l_x_ ≤ 200 μm, and the scanning step size in the Y direction, l_y_ ≤ Y/3. In this work, the preferred values for l_x_/l_y_ are l_x_ = 70 μm and l_y_ = 160 μm, respectively.

#### 2.5.3. SERS Imaging Mode

The SERS imaging mode is set according to peak area, and the integration range of the spectrum is 1300–1350 cm^−1^.

Using this method, the SERS image of the LFIA strip’s T line can be analyzed to make a visual determination of whether the sample is positive or negative for SARS-CoV-2. This methodology provides a robust means for signal recognition, leveraging both image-based analysis and Raman signal intensity variations.

### 2.6. SERS Image Identification Based on Residual Neural Network

The ResNet concept was first proposed by Kaiming He [14]. The core idea of a residual network is to introduce the ‘shortcut connection’ (residual block) to enable the network to learn the identity mapping. Compared to ordinary networks, it adds a short-circuit mechanism between every two or three layers, thus solving the model degradation problem in deep networks and improving the overall performance of the deep neural network [15]. The architecture of the ResNet model developed in this study (ResNet-18) is described in detail below.

#### 2.6.1. Overview of ResNet-18 Architecture

Depth and Composition: ResNet-18 is a deep convolutional neural network comprising 18 layers, including 17 convolutional layers and 1 fully connected layer. Input Layer: The network accepts RGB images of dimensions 3 × 224 × 224 (it can accept images of other sizes as well), which are the input representations of the SERS images. Convolutional Layers and ReLU Activation: The convolutional layers are responsible for extracting local features from the input images, with ReLU (Rectified Linear Unit) activation applied after each convolution to introduce non-linearity into the model. Residual Blocks: The network includes 8 residual blocks, each consisting of two convolutional layers with an identity-based skip connection. These residual blocks mitigate the vanishing and exploding gradient problems, allowing the network to effectively learn deeper features. Average Pooling Layer: The global average pooling layer transforms the final feature maps into a one-dimensional vector by computing the average of each feature map, thereby reducing the spatial dimensions while retaining essential information. Fully Connected Layer: The one-dimensional vector output from the pooling layer is fed into a fully connected layer with an output size of 1000 neurons, which serves as the classifier. Output Layer: The output from the fully connected layer is passed through a Softmax activation function, producing a probability distribution across 1000 classes.

#### 2.6.2. SERS Image Classification:

Based on the trained ResNet-18 architecture, SERS images obtained are classified into ‘Negative’ or ‘Positive’ categories. The classification is performed by analyzing the imaging characteristics defined in the previous section, which correspond to the presence or absence of viral antigens at the T line of the LFIA strip.

#### 2.6.3. Model Training and Testing:

Through the above methods, we tested a total of 187 samples. These included 89 positive samples with antigen concentrations ranging from 100 pg/mL to 2.5 pg/mL, as well as 98 negative samples (the negative samples were collected by the Shanghai Municipal Center for Disease Control and Prevention and came from different populations, and they were confirmed as negative through RT-PCR). The collected SERS images were randomly divided into a training set and a testing set in a 6:4 ratio for developing and testing the model. The model was implemented based on Pytorch. The network parameters were optimized based on the training set using the 5-fold cross-validation method. We employed the Adam optimizer with a batch size of 20 and a learning rate of 0.001, mainly optimizing the epochs of the model. The performance of the model on unseen data was evaluated using the testing set to ensure its generalization ability.

## 3. Results and Discussion

### 3.1. Characterization of Au NSs-DTNB-SiO_2_ Immunoprobes

**Figure 2:**
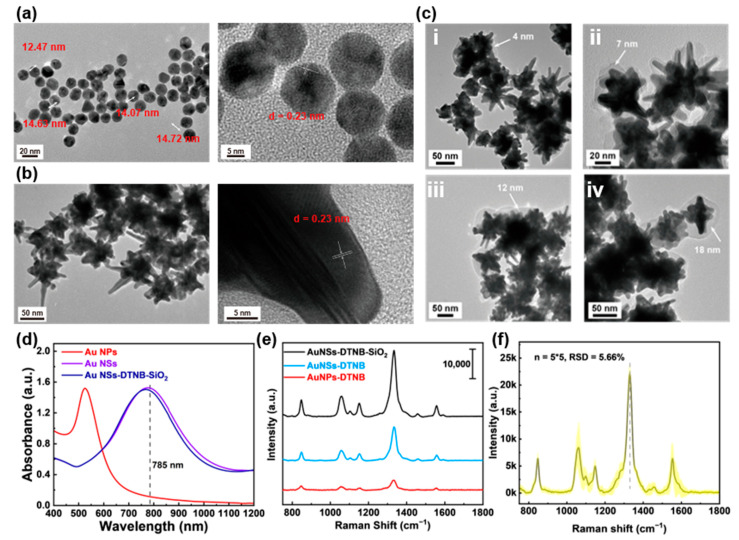
Characterization of Au NSs-DTNB-SiO_2_ immunoprobes. (**a**) TEM and HRTEM image of Au seeds with size 12–15 nm. (**b**) TEM and HRTEM image of Au NSs with size 50–100 nm. (**c**) TEM images of Au NSs coated with SiO_2_ shells of different thicknesses. (**d**) Absorption spectra of Au seeds, Au NSs, and SiO_2_-DTNB-Au NSs. (**e**) Raman spectra of DTNB enhanced by different nanoparticles. (**f**) The SERS stability of Au NSs. Note: 5 × 5 means 5 batches, with 5 measurements in each batch.

In our previous work, Au NSs demonstrated excellent SERS activity and were successfully employed as immunoprobes in SERS-LFIA technique [13]. As illustrated in Figure 2a, the Au seed particles (~14 nm in diameter) exhibit exposed (111) crystalline surfaces. Subsequent reduction in HAuCl_4_ by AA in the presence of AgNO_3_ promotes preferential growth along the (111) facets [16], yielding the final Au NSs (Figure 2b). These nanostructures feature a ~50 nm core and a pronounced spiky morphology, which is critical for SERS enhancement. To stabilize the probe molecules (DTNB) on the Au NSs surface, we encapsulated the SiO_2_ shell, forming a core–shell structure. Briefly, a controlled ratio of DTNB and the coupling agent (MPTS) was conjugated to the Au NSs via Au-S bonds. TEOS was then hydrolyzed under optimized pH conditions to deposit a tunable SiO_2_ layer. Figure 2c shows Au NSs-DTNB-SiO_2_ nanostructures synthesized with varying TEOS volumes (2–10 μL). TEM analysis confirmed the uniform SiO_2_ coatings with the thicknesses proportional to the TEOS amount: 4 nm (2 μL), 7 nm (4 μL), 12 nm (6 μL), and 18 nm (10 μL). Too little TEOS leads to incomplete coating of Au NSs, while too much TEOS causes agglomeration of nanoparticles. Therefore, we chose the Au NSs-SiO_2_ nanostructure with a 7 nm SiO_2_ shell layer for the subsequent experiments because it can balance the probe protection and the continuous ‘hot spot’ intensity. We measured the absorption spectrum of the nanoparticles, and the results are shown in Figure 2d. It can be seen that when the Au seeds grow into Au NSs, their maximum absorption peak shifts from the original ~520 nm to ~780 nm. The connection of DTNB and the coating of SiO_2_ have no significant effect on their LSPR properties. The Au NSs-SiO_2_ core–shell design offers dual advantages: (1) it shields DTNB from degradation or displacement; (2) it optimizes interparticle spacing, stabilizing plasmonic coupling and enhancing SERS signals [17]. Figure 2e displays the comparison results of the SERS performance of AuNPs, uncoated Au NSs, and SiO_2_-coated Au NSs. Au NSs outperformed AuNPs, while SiO_2_-coated Au NSs further amplified the DTNB signal, confirming that the SiO_2_ layer enhances both probe stability and SERS sensitivity. Furthermore, we conducted a test on the stability of the SERS substrate. The results are shown in Figure 2f. As can be seen, for the 5 batches, each batch was measured 5 times, and the relative standard deviation (RSD) was only 5.66%, indicating the excellent stability of this SERS substrate.

### 3.2. Performance Evaluation of Au NSs-DTNB-SiO_2_ Immunoprobe-Based SERS-LFIA Strips

To functionalize the Au NSs-DTNB-SiO_2_ probes for SARS-CoV-2 nucleoprotein (NP) detection, the SiO_2_ surface was aminated by using APTES to form Au NSs-DTNB-SiO_2_-NH_2_ probes. Antibodies were then covalently conjugated via EDC/NHS chemistry, followed by blocking of residual active sites. This covalent immobilization strategy was selected over electrostatic adsorption to enhance assay sensitivity [18].

**Figure 3:**
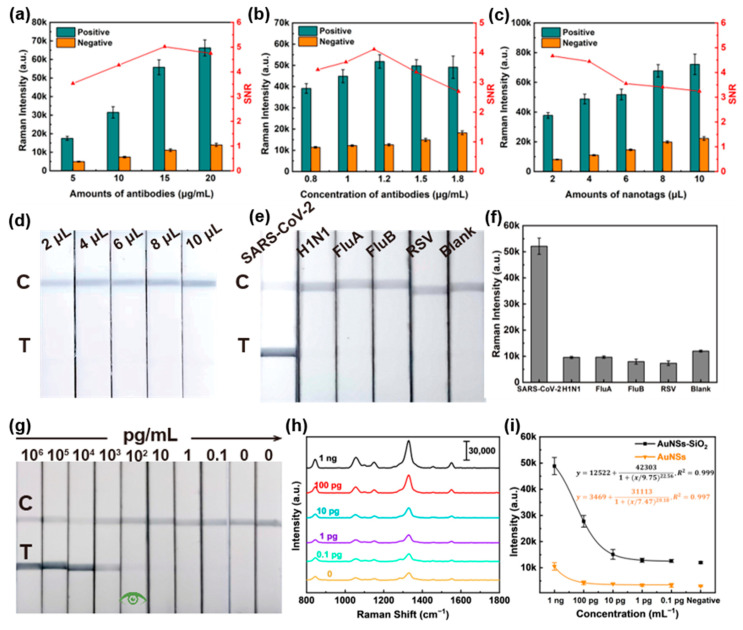
Performance evaluation of Au NSs-DTNB-SiO_2_ immunoprobe-based SERS-LFIA strips. (**a**–**c**) Optimization of assay conditions. (**d**) Photographs of the Au NSs-DTNB-SiO_2_ immunoprobe-based SERS-LFIA strips at different immunoprobe doses. (**e**) Visualization results of Au NSs-DTNB-SiO_2_ immunoprobe-based SERS-LFIA strip specificity assays for H1N1, Influenza B virus, Influenza A virus, and HRSV recombinant proteins. (**f**) Intensity of the Raman spectrum at the corresponding T line in (**e**) around 1330 cm^−1^. (**g**) Visualization sensitivity of the Au NSs-DTNB-SiO_2_ immunoprobe-based SERS-LFIA strips. (**h**) Raman spectra of the corresponding test strips in (**g**). (**i**) Fitting curves of Raman intensity around 1330 cm^−1^ for different concentrations of SARS-CoV-2 NPs detected by SERS-LFIA strips based on Au NSs-DTNB-SiO_2_ and Au NSs-DTNB immunoprobes.

This work evaluated the effects of various factors on the LFIA strip’s performance to identify optimal conditions for SARS-CoV-2 detection, such as the amount of capture antibody on the probe and the detection antibody at the T line and the quantity of the probe. Firstly, the optimal assay conditions were obtained by optimizing the concentration of the capture antibody on the Au NSs-DTNB-SiO_2_ nanoprobe (varied from 5 to 20 μg/mL) with the constant amount of immunoprobe. Then, 1 ng/mL of SARS-CoV-2 NP and blank PBS were applied as positive and negative samples. The SERS-LFIA strips were Raman detected through a 12 min immunochromatographic reaction and drying process. Based on the signal-to-noise ratio results (Figure 3a), the optimal capture antibody concentration was determined to be 15 μg/mL. Similarly, the optimal amount of detection antibody at the T line was investigated. Figure 3b shows that increasing the detection antibody amount can improve the signal-to-noise ratio, while excessive antibody levels can clog the NC membrane and reduce the ratio. Therefore, the optimal concentration of detection antibody was determined to be 1.2 mg/mL. Regarding the quantity of immunoprobe, although using a smaller quantity of immunoprobe can improve the signal-to-noise ratio, balancing sensitivity is crucial. Therefore, the optimal amount of immunoprobe was set at 4 μL to ensure both strong signal and sufficient sensitivity (Figure 3c). Furthermore, research on specificity and anti-interference indicated that the SERS-LFIA strips exhibited high specificity for SARS-CoV-2 NP. Even at increasing immunoprobe doses, no nonspecific T line signals were observed (Figure 3d). Cross-reactivity testing against H1N1, FluA, FluB, and HRSV antigens confirmed negligible interference, with Raman intensities indistinguishable from negative controls (Figure 3e,f).

Subsequently, the sensitivity of the test strips was evaluated. As shown in Figure 3g, the visual LOD of the Au NSs-DTNB-SiO_2_ immunoprobe-based SERS-LFIA strips for SARS-CoV-2 NP was 100 pg/mL. With respect to the samples without visible staining, Raman analysis results in Figure 3h show that the intensity of Raman signals in the T line gradually decreased with decreasing antigen concentration. The calibration curve of SERS intensity versus antigen concentration around 1330 cm^−1^ is applied to determine the theoretical LOD. In Figure 3i, the data show a strong dynamic relationship between SERS intensity and antigen concentration with correlation coefficients (R^2^) of 0.999 and a calculated LOD of 1.8 pg/mL (the cutoff values were set at the blank sample strength plus three times standard deviation). In addition, the Au NSs-DTNB immunoprobe-based SERS-LFIA strips were adopted for performance comparison, and the theoretical LOD was determined to 46 pg/mL. The above results illustrate the sensitivity advantage of the Au NSs-DTNB-SiO_2_ immunoprobe-based SERS-LFIA strips.

### 3.3. Recognition of T Line Signals Based on the Distribution of Nanoprobes

**Figure 4:**
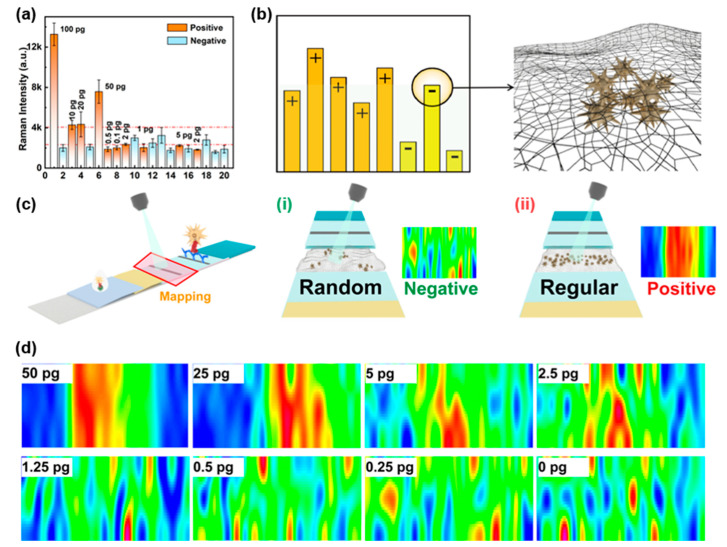
Signal analysis of SERS-LFIA strips based on a portable Raman spectroscopy. (**a**) SERS intensity results of 20 simulated blind samples. (**b**) Mechanism of false positive signals in negatives. (**c**) Principles of distribution-based signal discrimination: (**i**) negative (random probe distribution), (**ii**) positive (aligned distribution). (**d**) SERS imaging of samples at varying concentrations.

In current SERS-LFIA techniques, intensity-based signal identification remains the most common method for determining positive or negative results [19,20,21,22]. While our previous results demonstrate that this approach can achieve good sensitivity, balancing specificity and sensitivity in practical applications remains challenging [23,24]. To evaluate real-world performance, we conducted a simulated blind test using portable Raman spectroscopy on 20 randomized samples (10 negative samples and 10 SARS-CoV-2 NP samples ranging from 0.1 pg/mL to 100 pg/mL). As shown in Figure 4a, when using average signal intensity as the judgment criterion, four negative samples (Samples 10, 12, 13, and 18) exhibited anomalously high signals, forcing us to set the positive/negative threshold at 10 pg/mL or higher to avoid false positives. Without these outliers, the threshold could be lowered to 2–5 pg/mL, highlighting the limitations of intensity-based methods. The reasons for this phenomenon are mainly originated from the previously mentioned inhomogeneity of the NC membrane and the randomness of SERS sampling. As illustrated in Figure 4b, while positive samples generally show higher average signal intensities, sporadic aggregation of probes in negative samples can produce signals exceeding those of true positives. Especially at low concentrations of the analyst, if these anomalous signals happen to be collected during the test, the negative/positive discrimination of the results will inevitably be affected.

To address this challenge, we developed a novel signal recognition method that analyzes the spatial distribution of probes rather than relying solely on intensity. This approach involves rapid large-area scanning around the T line followed by Raman imaging. Negative samples exhibit only irregular, isolated signals without organized banding at the T line (Figure 4c-i), whereas positive samples show uniform probe distribution along the T line, forming clear bands analogous to conventional LFIA results (Figure 4c-ii). This distribution-based method effectively eliminates interference from anomalous signals caused by probe aggregation. Validation studies demonstrated that this approach consistently achieves a LOD of 2.5 pg/mL for SARS-CoV-2 NP (Figure 4d), significantly improving diagnostic accuracy compared to intensity-based methods. Through focusing on probe distribution patterns rather than absolute signal intensity, this strategy overcomes a critical limitation of conventional SERS-LFIA techniques while maintaining high sensitivity.

### 3.4. Automatic SERS Imaging Recognition Based on Residual Neural Networks

Although our optimized T line signal recognition scheme has significantly improved the reliability of SERS-LFIA strips based on Au NSs-DTNB-SiO_2_ immunoprobe, relying on human eye discrimination has great subjectivity and cannot be automated. To this end, the ResNet-18 image recognition model was applied to achieve automatic discrimination of results.

**Figure 5:**
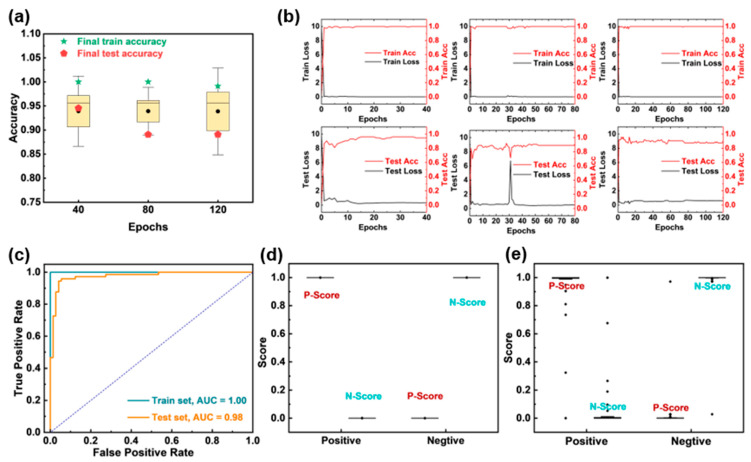
ResNet-18 model performance evaluation. (**a**) The 5-fold cross-validation results of the model under different epochs. (**b**) The loss rate and accuracy of the ResNet-18 model under different epochs of 40, 80, and 120. (**c**) ROC curves of the training and testing set. Scores of the (**d**) training and (**e**) testing sets.

To achieve rapid, highly sensitive, and specific automated detection of SARS-CoV-2, we constructed and trained the ResNet-18 model. Subsequently, we evaluated the performance of the model under different epochs using the five-fold cross-validation method. As shown in Figure 5a,b, considering the accuracy of the model and the training efficiency, we finally determined the epochs to be 40. Based on this, we verified the performance of the model on the ROC curves and score distributions in the training set and test set. Figure 5c shows that the area under the curve (AUC) for the training and testing sets reached 1.00 and 0.98, respectively, indicating the excellent discriminative ability. The P-scores (positive samples) and N-scores (negative samples) were well separated in both training and testing sets (Figure 5d,e). The confusion matrices (Table 1) indicate the high sensitivity of 100% and 91.43%, as well as the excellent specificity of 100% and 97.37% for the training and testing sets.

**Figure 6:**
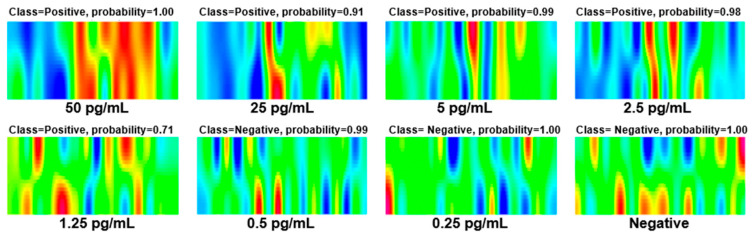
Experimental results of simulated human sample: Nasopharyngeal swabs from healthy individuals with different concentrations of SARS-CoV-2 NPs.

To further evaluate the generalization of this method, we conducted a simulated human experiment by adding SARS-CoV-2 NPs of different concentrations to the nasopharyngeal swabs of healthy individuals. The test results are shown in Figure 6. It can be seen that for samples with an added concentration of 2.5 pg/mL or above, the test results were all positive, and the probabilities were all above 0.9. The 1.25 pg/mL sample was also detected as positive, but the probability was only 0.71. Samples with an added concentration below 1.25 pg/mL could not be detected, and negative results were also correctly identified. These results indicate that our method has good generalization and potential for clinical application.

Furthermore, the detection limits and detection times of various SARS-CoV-2 detection methods were compared to evaluate the practicability and advancement of the developed SERS-LFIA system. As shown in Table 2, the comparison results indicates that although the developed SERS-LFIA system is not proven to have the highest detection sensitivity, it eliminates the need for complex sample preparation, lengthy detection time, and specialized operators compared with other detection protocols. Furthermore, the SERS-LFIA system integrates detection and discrimination functions to realize automation, thus avoiding to some extent the errors caused by subjective factors, which is a unique advantage in clinical and field environments.

## 4. Conclusions

In this study, we developed an ultra-sensitive automated detection system that integrates SERS scanning imaging with a deep learning-based model (ResNet-18) for rapid and accurate SARS-CoV-2 diagnosis. The SERS-LFIA strips employ SiO_2_-coated Au NSs as the immunoprobes, achieving a visual LOD of 100 pg/mL and an exceptional theoretical Raman LOD of 1.8 pg/mL for SARS-CoV-2 NPs. To overcome the limitations of Raman intensity-based analysis, a novel signal identification method that uses ResNet-18 for SERS images near the T line was introduced to analyze the distribution pattern of the immunoprobes, which greatly reduces the influence of abnormal signals on the negative–positive discrimination. Through integrating the entire assay workflow, the system eliminates the need for specialized operators and delivers results within 10–15 min, making it a scalable POCT solution. Furthermore, the platform’s adaptability allows for the detection of other pathogens (e.g., influenza, HRSV, or emerging viruses) through modification of antibody-functionalized nanoprobes, offering a versatile tool for pandemic preparedness.

## Figures and Tables

**Table 1:** Confusion matrix based on the ResNet-18 model.

		Training Set (114 Samples)		Testing Set (73 Samples)	
		Original Class		Original Class	
		Positive	Negative	Positive	Negative
Predicted class	Positive	54	0	32	1
	Negative	0	60	3	37
Sensitivity		100.00%		91.43%	
Specificity		100.00%		97.37%	

**Table 2:** Comparison of the various SARS-CoV-2 detection methods.

Sensor Type	Immunoprobe	LOD (pg/mL)	Detection Time	Usability	Ref.
Electrochemical	PPG	42	Hours	labs	[5]
MeSA-eMeSA	screen-printed carbon electrodes	8890	10–15 min	labs	[25]
MIP systems	polypyrrole	51.2	Not mentioned	labs	[26]
PEC	CdS: Mn sensitized Bi_2_MoO_6_/In_2_S_3_ and NaYF_4_: Yb, Er for signal amplification	0.0036	Not mentioned	labs	[27]
SERS	Ti_3_C_2_T_x_@Ag	3.24	Not mentioned	labs	[28]
SERS	BP/ZIF-67	6400	~30 min	labs	[29]
LFIA	PEG-SeNP	10	1 min	POCT	[30]
catalytic colorimetric-LFIA	Fe_3_O_4_@MoS_2_@Pt	80	10–15 min	POCT	[7]
photothermal-LFIA		10	10–15 min		
SERS-LFIA	Ag/BP	0.5	10–15 min	POCT	[12]
SERS-LFIA	SiO_2_-Au NSs	1.8~2.5	10–15 min	Automated POCT	This work

## Data Availability

The dataset and code used in this research can be obtained by contacting the authors.

## References

[1] Peng W.Y., Wu H., Tan Y., Li M., Yang D.C., Li S. (2020). Mechanisms and treatments of myocardial injury in patients with corona virus disease 2019. Life Sci..

[2] Taleghani N., Taghipour F. (2021). Diagnosis of COVID-19 for controlling the pandemic: A review of the state-of-the-art. Biosens. Bioelectron..

[3] Xu L.Z., Li D.Y., Ramadan S., Li Y.B., Klein N. (2020). Facile biosensors for rapid detection of COVID-19. Biosens. Bioelectron..

[4] Tong H.Y., Cao C.Y., You M.L., Han S., Liu Z., Xiao Y., He W.H., Liu C., Peng P., Xue Z.R. (2022). Artificial intelligence-assisted colorimetric lateral flow immunoassay for sensitive and quantitative detection of COVID-19 neutralizing antibody. Biosens. Bioelectron..

[5] Du H., Dang X., Chen R., Li Y., Cui N., Yang H. (2024). A universal three-dimensional hydrogel electrode for electrochemical detection of SARS-CoV-2 nucleocapsid protein and hydrogen peroxide. Biosens. Bioelectron..

[6] Fang B.L., Xiong Q.R., Duan H.W., Xiong Y.H., Lai W.H. (2022). Tailored quantum dots for enhancing sensing performance of lateral flow immunoassay. Trac-Trends Anal. Chem..

[7] Xu M., Zhao S., Lin C., Li Y., Zhang W., Peng Y., Xiao R., Huang Z., Yang Y. (2024). Dual-Mode Lateral Flow Immunoassay Based on “Pompon Mum”-Like Fe3O4@MoS2@Pt Nanotags for Sensitive Detection of Viral Pathogens. ACS Appl. Mater. Interfaces.

[8] Khlebtsov B., Khlebtsov N. (2020). Surface-Enhanced Raman Scattering-Based Lateral-Flow Immunoassay. Nanomaterials.

[9] Langer J., Jimenez de Aberasturi D., Aizpurua J., Alvarez-Puebla R.A., Auguié B., Baumberg J.J., Bazan G.C., Bell S.E.J., Boisen A., Brolo A.G. (2020). Present and Future of Surface-Enhanced Raman Scattering. ACS Nano.

[10] Khlebtsov B.N., Bratashov D.N., Byzova N.A., Dzantiev B.B., Khlebtsov N.G. (2018). SERS-based lateral flow immunoassay of troponin I by using gap-enhanced Raman tags. Nano Res..

[11] Liu Z., Wang C., Zheng S., Yang X., Han H., Dai Y., Xiao R. (2023). Simultaneously ultrasensitive and quantitative detection of influenza A virus, SARS-CoV-2, and respiratory syncytial virus via multichannel magnetic SERS-based lateral flow immunoassay. Nanomed. Nanotechnol. Biol. Med..

[12] Lin C., Liu Z., Fang F., Zhao S., Li Y., Xu M., Peng Y., Chen H., Yuan F., Zhang W. (2023). Next-Generation Rapid and Ultrasensitive Lateral Flow Immunoassay for Detection of SARS-CoV-2 Variants. ACS Sens..

[13] Zhao S., Lin C.L., Xu M.M., Zhang W.D., Li D., Peng Y.S., Huang Z.R., Yang Y. (2024). Comprehensive SERS-LFIA Platform for Ultrasensitive Detection and Automated Discrimination of Chloramphenicol Residues in Aquatic Products. ACS Appl. Nano Mater..

[14] He K., Zhang X., Ren S., Sun J. Deep Residual Learning for Image Recognition. Proceedings of the 2016 IEEE Conference on Computer Vision and Pattern Recognition (CVPR).

[15] Xiao Q., Wang Y., Fan J., Yi Z., Hong H., Xie X., Huang Q.-a., Fu J., Ouyang J., Zhao X. (2024). A computer vision and residual neural network (ResNet) combined method for automated and accurate yeast replicative aging analysis of high-throughput microfluidic single-cell images. Biosens. Bioelectron..

[16] Wu H.-L., Chen C.-H., Huang M.H. (2008). Seed-Mediated Synthesis of Branched Gold Nanocrystals Derived from the Side Growth of Pentagonal Bipyramids and the Formation of Gold Nanostars. Chem. Mater..

[17] Li J.F., Huang Y.F., Ding Y., Yang Z.L., Li S.B., Zhou X.S., Fan F.R., Zhang W., Zhou Z.Y., Wu D.Y. (2010). Shell-isolated nanoparticle-enhanced Raman spectroscopy. Nature.

[18] Guo J.C., Chen S.Q., Guo J.H., Ma X. (2021). Nanomaterial Labels in Lateral Flow Immunoassays for Point-of-Care-Testing. J. Mater. Sci. Technol..

[19] Liu H.F., Dai E.H., Xiao R., Zhou Z.H., Zhang M.L., Bai Z.K., Shao Y., Qi K.Z., Tu J., Wang C.W. (2021). Development of a SERS-based lateral flow immunoassay for rapid and ultra-sensitive detection of anti-SARS-CoV-2 IgM/IgG in clinical samples. Sens. Actuators B-Chem..

[20] Law J.W.F., Ab Mutalib N.S., Chan K.G., Lee L.H. (2015). Rapid methods for the detection of foodborne bacterial pathogens: Principles, applications, advantages and limitations. Front. Microbiol..

[21] Li Z., Wang Y., Wang J., Tang Z., Pounds J.G., Lin Y. (2010). Rapid and Sensitive Detection of Protein Biomarker Using a Portable Fluorescence Biosensor Based on Quantum Dots and a Lateral Flow Test Strip. Anal. Chem..

[22] Roberts A., Chouhan R.S., Shahdeo D., Shrikrishna N.S., Kesarwani V., Horvat M., Gandhi S. (2021). A Recent Update on Advanced Molecular Diagnostic Techniques for COVID-19 Pandemic: An Overview. Front. Immunol..

[23] Hsieh W.Y., Lin C.H., Lin T.C., Lin C.H., Chang H.F., Tsai C.H., Wu H.T., Lin C.S. (2021). Development and Efficacy of Lateral Flow Point-of-Care Testing Devices for Rapid and Mass COVID-19 Diagnosis by the Detections of SARS-CoV-2 Antigen and Anti-SARS-CoV-2 Antibodies. Diagnostics.

[24] Abdelmalek S., Hamed W., Nagy N., Shokry K., Abdelrahman H. (2022). Evaluation of the diagnostic performance and the utility of stool antigen lateral immunochromatography assay. Heliyon.

[25] Fukana N., Park J., Silva Junior G.J., Malsick L.E., Gallichotte E.N., Ebel G.D., Geiss B.J., Dandy D.S., Bertotti M., Nacapricha D. (2025). Magnetophoretic slider assay for electrochemical detection of SARS-cov-2 nucleocapsid protein in nasal swab samples. Biosens. Bioelectron..

[26] Drobysh M., Ratautaite V., Brazys E., Ramanaviciene A., Ramanavicius A. (2024). Molecularly imprinted composite-based biosensor for the determination of SARS-CoV-2 nucleocapsid protein. Biosens. Bioelectron..

[27] Xu R., Wang H., Li Y., Gu J., Ren X., Ma H., Wu D., Wei Q. (2025). A sandwich-type photoelectrochemical immunosensor for the detection of SARS-CoV-2 N protein based on CdS:Mn sensitized Bi_2_MoO_6_/In_2_S_3_ and NaYF_4_:Yb, Er for signal amplification. Sens. Actuators B Chem..

[28] Chen J., Zhang X., Wang C., Wang S., Gu C., Zeng S., Jiang J., Jiang T., Wu K. (2025). In–situ self–reduction preparation of Ti3C2Tx/Ag on flexible PMMA chip for quantitative detection of SARS–CoV–2. Sens. Actuators B Chem..

[29] Xue D., Zhang J., Liu H., Gu C., Zhou X., Jiang T., Wu K. (2024). Hydrophilic-hydrophobic polymer strip with intrinsic signal and chemical-electromagnetic synergistic enhancement for non-metallic SERS-based identification of SARS-CoV-2 antigen. Sens. Actuators B Chem..

[30] Chen C., Duan S., Ji J., Wu M., Yang Z., Cai M., Xue M., Wang L., Chen R., Yaron S. (2025). Structured protein probes modified with selenium nanoparticle for 1-minute measurement of SARS-CoV-2 antigen. Biosens. Bioelectron..

